# PPAR*α* Targeting GDF11 Inhibits Vascular Endothelial Cell Senescence in an Atherosclerosis Model

**DOI:** 10.1155/2021/2045259

**Published:** 2021-02-25

**Authors:** Fangfang Dou, Beiling Wu, Jiulin Chen, Te Liu, Zhihua Yu, Chuan Chen

**Affiliations:** Shanghai Geriatric Institute of Chinese Medicine, Shanghai University of Traditional Chinese Medicine, Shanghai 200031, China

## Abstract

Atherosclerosis (AS) is a complex vascular disease that seriously harms the health of the elderly. It is closely related to endothelial cell aging, but the role of senescent cells in atherogenesis remains unclear. Studies have shown that peroxisome proliferator-activated receptor alpha (PPAR*α*) inhibits the development of AS by regulating lipid metabolism. Our previous research showed that PPAR*α* was involved in regulating the repair of damaged vascular endothelial cells. Using molecular biology and cell biology approaches to detect senescent cells in atherosclerosis-prone apolipoprotein E-deficient (*Apoe*^−/−^) mice, we found that PPAR*α* delayed atherosclerotic plaque formation by inhibiting vascular endothelial cell senescence, which was achieved by regulating the expression of growth differentiation factor 11 (GDF11). GDF11 levels declined with age in several organs including the myocardium, bone, central nervous system, liver, and spleen in mice and participated in the regulation of aging. Our results showed that PPAR*α* inhibited vascular endothelial cell senescence and apoptosis and promoted vascular endothelial cell proliferation and angiogenesis by increasing GDF11 production. Taken together, these results demonstrated that PPAR*α* inhibited vascular endothelial cell aging by promoting the expression of the aging-related protein GDF11, thereby delaying the occurrence of AS.

## 1. Introduction

The occurrence and development of atherosclerosis (AS) are closely related to endothelial dysfunction caused by endothelial cell aging. Many cardiovascular risk factors, such as hypertension, hyperlipidemia, and diabetes, can cause endothelial cell aging, leading to endothelial cell dysfunction and AS [1, 2]. Vascular endothelial cells are a semipermeable membrane barrier between the blood and subendothelial tissues which have sensing and secretion functions, and they produce effector molecules to regulate thrombosis, inflammation, vascular tone, and vascular reconstruction [3]. Aging impairs the function of endothelial cells, increases their permeability to lipoproteins and plasma components, reduces nitric oxide secretion, and increases intercellular cell adhesion molecule-1 (ICAM-1) and vascular cell adhesion molecule-1 (VCAM-1) secretion and nuclear transcription factor-*κ*B (NF-*κ*B) expression, favoring a proinflammatory and proapoptotic state [4–6]. Studies have shown that telomere length of endothelial cells decreases with age, and the degree of shortening is more pronounced in sites prone to AS [7]. Animal experiments have found that vascular branches or branch points were susceptible to hemodynamic shearing and stretching changes, resulting in chronic damage to endothelial cells, and endothelial cells at these sites improve cell turnover to maintain their own morphology and function and are prone to senescence, which may cause atherosclerotic plaque formation [8]. Platelet-derived growth factor (PDGF) secreted by damaged endothelial cells promotes smooth muscle cell chemotaxis and proliferation. Inflammatory factors released by damaged endothelial cells recruit a large number of monocytes and polymorphonuclear neutrophils. ICAM-1 and VCAM-1 secreted by damaged endothelial cells promote adhesion of monocytes. Therefore, endothelial dysfunction caused by endothelial cell aging is likely the initiating factor of AS [9].

Peroxisome proliferator-activated receptor alpha (PPAR*α*) is a nuclear receptor that regulates the transcription of various target genes by binding to ligands (such as fatty acids and fibrates), thereby affecting lipid metabolism, inflammatory response, and AS; PPAR*α* also plays a role in maintaining blood sugar stability and enhancing tissue sensitivity to insulin [10]. In addition, PPAR*α* not only prevents the development of AS but also stabilizes atherosclerotic plaques. Moreover, it even reverses the development of atherosclerotic plaques and prevents the occurrence of acute cardiovascular events. Studies have shown that PPAR*α* directly inhibits the accumulation of monocytes to vascular endothelial cells and transformation into macrophages. It also inhibits the proliferation and migration of vascular smooth muscle cells, inhibits the formation of foam cells, and reduces the plaque instability by directly acting on the arterial wall [11]. PPAR*α* inhibits gene transcription related to inflammatory response and slows down plaque formation by downregulating the expression of inflammatory factors [12]. PPAR*α* inhibits thrombin-induced synthesis of endothelin-1 by activating protein-1-mediated signaling pathways and improves vascular function [13]. Clinical experiments have found significantly reduced levels of plasma interleukin-6 (IL-6), interferon-*α* (IFN-*α*), and C-reactive protein (CRP) after taking the PPAR*α* agonist (fibrate) in patients with coronary heart disease confirmed by angiography [14]. Our previous research confirmed that PPAR*α* promotes the repair of endothelial cell injury by upregulating CCL2 expression in human umbilical vein endothelial cells [15]. Some research reported that Tongxinluo protects diabetic hearts against ischemia/reperfusion injury by activating Angptl4-mediated restoration of endothelial barrier integrity via the PPAR*α* pathway [16]. In addition, PPAR*α* agonists induce nitric oxide synthase (NOS) expression, which leads to increased NO production in vascular endothelial cells, suggesting a vasculoprotective effect [17]. PPAR*α* has also been implicated in the regulation of redox responses in the endothelium, and increasing evidence suggests that excessive oxidative stress is a major contributor to endothelial dysfunction [18]. PPAR*α* induces the expression of the cytosolic Cu, Zn-SOD (SOD1) and attenuates the induction of p22 and p47phox subunits of the superoxide-producing nicotinamide adenine dinucleotide phosphate oxidase (NOX) in primary endothelial cells [19]. However, it is still unclear whether PPAR*α* delays the occurrence of AS by inhibiting vascular endothelial cell aging. Our research found that PPAR*α* inhibited the aging of vascular endothelial cells by promoting the expression of aging-related protein growth differentiation factor 11 (GDF11), thereby delaying the occurrence of AS.

## 2. Materials and Methods

### 2.1. Animals

Eighty adult male *Apoe^−/−^* C57BL/6 mice (8 weeks old, 20–25 g) were used for the following experiments: hematoxylin and eosin (HE) staining, Masson staining, beta galactosidase (*β*-gal) staining, immunofluorescence staining (*n* = 20), transmission electron microscopy studies (*n* = 20), real-time PCR assay (*n* = 20), and western blot assay (*n* = 20). *Apoe^−/−^* mice were obtained from the model animal laboratories of Charles River, Beijing, China. Experimental animals were divided into four groups: *Apoe^−/−^* mice on a normal diet (control group), *Apoe^−/−^* mice on a high-fat diet (model group), pemafibrate-treated *Apoe^−/−^* mice on a high-fat diet (PPAR*α* agonist group), and GW6471-treated *Apoe^−/−^* mice on a high-fat diet (PPAR*α* antagonist group). Pemafibrate and GW6471 (MedChemExpress, USA) were administered via gavage through a stomach tube for 3 months. Pemafibrate was dissolved in dimethyl sulfoxide (DMSO) and administered at a dosage of 0.03 mg/kg mouse/day [20]. GW6471 was also dissolved in DMSO and administered at 20 mg/kg mouse/day [21]. All *Apoe^−/−^* mice were kept in the SPF-grade animal facility at the animal center of the Shanghai University of Traditional Chinese Medicine. *Apoe^−/−^* mice were housed in a temperature-controlled environment and maintained on a light/dark cycle of 12 hours/12 hours, and the room temperature was maintained at 24°C with relative humidity of 50%–60%. All drug gavages and tissue extractions were approved by the Animal Care Committee for the use of laboratory animals at the Shanghai University of Traditional Chinese Medicine.

### 2.2. Hematoxylin and Eosin Staining

*Apoe^−/−^* mice were deeply anesthetized by intraperitoneal injections of sodium pentobarbital (80 mg/kg) and with 20 mL of 0.9% saline, followed by 50 mL of 4% paraformaldehyde in cold 0.01 M phosphate-buffered saline (PBS, pH 7.4). After perfusion, the left ventricular outflow tract tissue was carefully removed and postfixed for additional 24 hours in the same fixation solution at 4°C. Paraffin sections of the left ventricular outflow tract tissues of *Apoe^−/−^* mice were dewaxed with xylene (Sinopharm, China), hydrated with gradient ethanol (100%, 95%, and 75%) (Sinopharm, China), stained with hematoxylin solution (Beyotime, China) for 5–10 min, and immersed in tap water for 10 min to remove the excess staining solution. Then, the sections were stained with eosin solution (Beyotime, China) for 2 min, dehydrated with gradient ethanol (75%, 95%, and 100%), made transparent with xylene for 5 min, and covered with neutral balsam (Beyotime, China). The tissue sections were observed under a microscope (Olympus, Japan), where the nuclei were stained blue and the cytoplasm was pink or red.

### 2.3. Masson's Trichrome Staining

Masson's trichrome staining is a classical method of staining connective tissue and collagen fibers, where muscle fibers are stained red and collagen fibers are green or blue (Solarbio, China). Briefly, paraffin sections of the left ventricular outflow tract tissues of *Apoe^−/−^* mice were dewaxed with xylene, hydrated with gradient ethanol, dyed with the prepared Weigert's iron hematoxylin staining solution for 5–10 min, differentiated with acidic ethanol differentiation solution for 5–15 s, and washed with water. The sections were returned to blue for 3–5 minutes with Masson's blue solution before washing with water, dyed with Ponceau magenta dyeing solution for 5–10 min, stained with aniline blue solution for 1–2 min after washing with weak acid working solution and phosphomolybdic acid solution for 1–2 min, dehydrated with gradient ethanol, made transparent with xylene, covered with neutral balsam after washing with weak acid working solution, and observed under a microscope.

### 2.4. Transmission Electron Microscopy

*Apoe^−/−^* mice were anesthetized by intraperitoneal injections of sodium pentobarbital (80 mg/kg) and perfused with 20 mL of 0.9% saline, followed by 50 mL of 3% glutaraldehyde. Plaque tissues of the aorta were taken under a dissecting microscope and cut to 1 mm^3^. The excised aortic plaque tissues were immersed in 3% glutaraldehyde for 3 days. Aortic plaque tissues were postfixed in 2% osmium tetroxide for 2 h, stained with 1% uranyl acetate for 60 min before rinsing in water, and dehydrated in graded concentrations of ethanol. Subsequently, the tissues were embedded in 812 embedding resin (VWR-Bie and Berntsen A/S, Herlev, Denmark) and polymerized at 60°C for 24 hours. Sections (70 nm thick) were collected on copper grids coated with 2% parlodion and stained with Reynold's lead citrate. Imaging was conducted with a JEM-1011 transmission electron microscope (JEOL, Japan).

### 2.5. Beta Galactosidase Staining

The dewaxing and rehydration process of the paraffin section of left ventricular outflow tract tissue of *Apoe^−/−^* mice was performed as previously described. The cultured human umbilical vein endothelial cells (HUVECs) were fixed with 4% paraformaldehyde before washing in PBS for beta galactosidase (*β*-gal) (Beyotime, China) staining. Briefly, the prepared *β*-gal staining solutions were added to tissue sections or cell culture dishes and incubated in a 37°C incubator overnight. Tissue sections were covered with 75% glycerol, and the tissues and cells were observed directly under the microscope.

### 2.6. Immunofluorescence Double Labeling Staining

Dewaxed and hydrated paraffin sections of the left ventricular outflow tract tissue of *Apoe^−/−^* mice were boiled with sodium citrate at 100°C for 30 min to repair antigens. The sections were blocked with a blocking solution (Beyotime, China) at room temperature for 30 min. They were exposed to the diluted primary antibody solution containing a primary antibody (CD31/p16 or CD31/GDF11). A CD31 rabbit monoclonal antibody (mAb) (1 : 200) was purchased from Cell Signaling Technology (CST, USA), while p16 mouse mAb (1 : 200) and GDF11 mouse mAb (1 : 100) were purchased from Abcam. The tissue sections were incubated with primary antibodies at 4°C for 24 h and washed with PBS for three times after discarding the primary antibody. Subsequently, the sections were incubated with a secondary antibody (goat anti-rabbit IgG Alexa Fluor 546 and/or goat anti-mouse IgG Alexa Fluor 488, 1 : 500, Thermo Fisher Scientific, USA) at 37°C for 1 h and washed with PBS three times before covering with 75% glycerol containing 1% DAPI (Beyotime, China). Finally, the tissue sections were observed under a fluorescence microscope (Olympus, Japan).

### 2.7. Cell Culture

EA.hy926 cells (Cell Bank, Shanghai Institute for Biological Science, Shanghai, China) and primary HUVECs were separately cultured in Dulbecco's modified Eagle's medium (DMEM; Thermo Fisher Scientific, MA, USA) supplemented with 10% heat-inactivated fetal bovine serum (FBS; Thermo Fisher Scientific, USA) or endothelial cell growth medium-2 (EGM-2, Lonza, USA) at 37°C in a humidified atmosphere of 95% air and 5% CO_2_. EA.hy926 cells were used for RNA and protein extraction, luciferase assay, and electrophoretic mobility shift assay (EMSA), while HUVECs were used for the *β*-gal staining assay, ki67 staining assay, terminal deoxyribonucleotide transferase-mediated dUTP-digoxigenin end labeling (TUNEL) staining assay, and angiogenesis assay. Cells treated with PBS were a control group, while cells treated with 200 *μ*g/mL of oxidized low-density lipoprotein (ox-LDL) (Yuanye Biotechnology, China) for 24 h were a model group. PPAR*α* and GDF11 overexpressed or knockdown lentivirus was purchased from Shanghai Novobio Biotechnology Co., Ltd. The virus titer was 1 × 10^8^ TU/mL. Cells were seeded in cell culture dishes or plates, and the virus infection was realized when the density of cells reached more than 90%. According to the number of infected cells, viruses and virus infection enhancement solution HitransG A were added. The cells were cultured at 37°C for 24 h before the culture media were refreshed, according to the number of green fluorescent protein-expressing cells. The rate of cell infection was examined 72 h after the infection. The cell infection rate greater than 90% could be used for functional experiments. Cell groups with overexpressed PPAR*α* or GDF11 were labeled as PPAR*α* ov or GDF11 ov, respectively; cells with interfered PPAR*α* or GDF11 were labeled as PPAR*α* RNAi or GDF11 RNAi, respectively. PPAR*α* and GDF11 ov groups or RNAi groups were all accompanied by model condition. GDF11 RNAi and PPAR*α* agonists were only added to ox-LDL treatment cells.

### 2.8. Real-Time PCR Assay

Total RNA was extracted from aortic tissue of *Apoe^−/−^* mice or the cultured cells. The SYBR Green Real-Time PCR Master Mix Kit (Toyobo, Japan) was used for all polymerase chain reactions (PCR). Two microliters of cDNA, 0.4 *μ*mol forward and reverse primers, 1 *μ*L SYBR Green Real-Time PCR Master Mix, and appropriate amount of distilled water made up the 20 *μ*L reaction system for target gene amplification. The cycle steps of real-time PCR included the following: 95°C for 60 s and 40 PCR cycles (95°C denaturing for 5 s and 60°C annealing and extension for 30 s). In the amplification curve and dissolution curve, a region where the fluorescence increased exponentially was observed for analyzing the authenticity of the acquired data. The comparative threshold cycle (ΔC_T_) method was used to quantify target mRNAs, and GAPDH was used as an internal reference gene. The primers used for reaction were as follows: p16 (sense 5′-CGCAGGTTCTTGGTCACTGT-3′ and antisense 5′-TGTTCACGAAAGCCAGAGCG-3′), p66 (sense 5′-AAGTACAACCCACTTCGGAATG-3′ and antisense 5′-GAAAGAAGGAACACAGGGTAGTC-3′), junD (sense 5′-GGCGGGATTGAACCAGGG-3′ and antisense 5′-AGCCCGTTGGACTGGATGA-3′), TNF*α* (sense 5′-TTGGAACTTGGAGGGCTAGG-3′ and antisense 5′-CACTAAGGCCTGTGCTGTTC-3′), IL-1*β* (sense 5′-CTCTCTCCTTTCAGGGCCAA-3′ and antisense 5′-GCGGTTGCTCATCAGAATGT-3′), IL-6 (sense 5′-AGACAGCCACTCACCTCTTC-3′ and antisense 5′-AGTGCCTCTTTGCTGCTTTC-3′), C-X-C motif ligand 10 (CXCL10) (sense 5′-CCAAGTGCTGCCGTCATTTTC-3′ and antisense 5′-GGCTCGCAGGGATGATTTCAA-3′), plasminogen activator inhibitor-1 (PAI-1) (sense 5′-TCTGGGAAAGGGTTCACTTTACC-3′ and antisense 5′-GAGGGAGAGAAG-3′), matrix metalloproteinase-3 (MMP3) (sense 5′-GGCCTGGAACAGTCTTGGC-3′ and antisense 5′-TGTCCATCGTTCATCATCGTCA-3′), and GAPDH (sense 5′-ACCCAGAAGACTGTGGATGG-3′ and antisense 5′-TCAGCTCAGGGATGACCTTG-3′). All primers were synthesized by Sangon Biotech (Shanghai) Co., Ltd.

### 2.9. Western Blot Assay

Total protein was extracted from the aortic tissue of *Apoe^−/−^* mice or the cultured cells. The BCA protein assay kit (Beyotime, China) was used for total protein concentration determination. Namely, 30 *μ*g of total proteins was subjected to SDS-PAGE, and the proteins were transferred to 0.22 *μ*m aperture nitrocellulose filter membranes (PVDF membranes, Millipore, USA). The PVDF membrane was blocked with 2% bovine serum albumin (BSA) for 30 min at room temperature and incubated with the primary antibody at 4°C overnight. It was washed with 0.1 M PBS for three times, followed by incubation with secondary antibodies at room temperature for 2 h after PBS washing. Finally, the ECL chemiluminescence detection kit was used for protein quantification. Primary polyclonal antibodies included anti-p16 (1 : 1000, CST, USA), anti-p66shc (1 : 2000, CST, USA), anti-junD (1 : 1000, Boster, China), anti-GDF11 (1 : 1000, CST, USA), anti-GAPDH (1 : 2000, Boster, China), and anti-*β*-actin (1 : 5000, CST, USA). Secondary horseradish peroxidase-conjugated goat anti-rabbit and goat anti-mouse immunoglobulin- (Ig-) G antibodies (1 : 2000) were purchased from Cell Signaling Technology.

### 2.10. Luciferase Reporter Assay

EA.hy926 cells seeded into six-well cell culture plates were infected with the lentivirus GDF11 plasmid. Human GDF11 gene promoter reporter gene plasmids include PDS131_psiCHECK-2 blank vector (6273 bp), PDS131_psiCHECK-2-GDF11 wild type (wt, gene synthesis of the GDF11 gene 5′UTR 195 bp and promoter region 1500 bp and subcloning into the reporter vector), and PDS131_psiCHECK-2-GDF11 mutant (mut, deletion mutation of “AAGCCCCAG” in the promoter region of the wild-type vector GDF11 gene) (Novobio Biotechnology Co. Ltd., Shanghai, China). The dual-luciferase reporter assay kit (Beyotime, China) was used to detect the luciferase activity in each group after 72 h from the transfection. The microplate reader (Molecular Devices, USA) was used to detect the corresponding value of the fluorescence emitted by the firefly luciferase-inducing substrate (FL). Then, the corresponding value of the fluorescence released by the Renilla luciferase-inducing substrate (RL) was detected. The ratio of the value of FL to RL was measured for each sample as the relative luciferase activity of the reporter gene.

### 2.11. Electrophoretic Mobility Shift Assay

Total nuclear proteins were extracted from the cultured cells using a cell fractionation kit (CST, USA). Namely, 0.5× TBE nondenaturing gel (5.5%) was equipped for protein gel electrophoresis. At first, preelectrophoresis (120 V) was performed for 30 min. Protein and corresponding probe (the probe labeled with biotin) were mixed and placed at room temperature for 20 min. Sample buffer was added to the sample mixture (protein and probe) for electrophoresis (180 V, 60 min). Subsequently, probe-labeled protein was transferred to the PVDF membrane, and the transferred PVDF was crosslinked at 20 cm under the ultraviolet lamp for 20 min. The nylon membrane after crosslinking was blocked with a blocking solution at room temperature for 20 min and was incubated with a streptavidin-HRP conjugate antibody (1 : 2000) for 30 min. The membrane was equilibrated with the equilibration solution for 5 min. The ECL chemiluminescence detection kit was used to detect the signaling. The probes included PPAR*α* probe (5′-TCACCCTTCCCCCAGTGGCC-3′-biotin), cold probe (5′-TCACCCTTCCCCCAGTGGCC-3′), and mutant probe (5′-TCACCCTTCCCCCAACGGCC-3′-biotin), loading samples including negative control (labeled probe only), control (protein only), PPAR*α* probe (protein plus labeled probe), cold probe (protein plus unlabeled probe), and mutant probe (protein plus mutant probe plus unlabeled probe).

### 2.12. Cell Proliferation Assay

HUVECs treated with ox-LDL or transfected with GDF11 plasmids were fixed with 4% paraformaldehyde twice for 10 min at room temperature. The fixed cells were rinsed with PBS and blocked with a blocking solution containing 5% normal goat serum. A primary ki67 antibody (Beyotime, China) was used for detecting the proliferation of cells. Goat anti-rabbit IgG Alexa Fluor 546 was used to display red marked cells as proliferating HUVECs, while DAPI was used to confirm the nuclei. The number of ki67-positive cells was counted under a microscope and compared between the groups.

### 2.13. Angiogenesis Assay

HUVECs treated with ox-LDL or transfected with GDF11 plasmids were seeded into the Matrigel® basement membrane- (BD, USA) coated 96-well plates. HUVECs were incubated for 48 h with EGM-2 culture medium including 20 ng/mL VEGF165 (Lonza, USA). Angiogenesis was observed under an optical microscope, and the number of branch points in each group was used for comparison between groups.

### 2.14. TUNEL Staining

The TUNEL (Beyotime, China) assay was used to detect apoptotic cells. HUVECs seeded in 24-well plates were fixed with 4% paraformaldehyde. Then, 0.3% Triton X-100 diluted in PBS was added to the cells and incubated for 5 min at room temperature. TUNEL solution was added to the cells and incubated at 37°C for 60 min in the dark. The cells were covered with 75% glycerol and observed under a fluorescence microscope. The number of green fluorescent labeled cells in each group was compared between the groups.

### 2.15. Statistical Analysis

Statistical analysis was performed using SPSS v. 18.0 software (SPSS Inc., USA). All data were presented as mean ± SD. One-way ANOVA and Tukey's *post hoct*-tests were used to determine the level of statistical significance. *P* < 0.05 was considered to have a statistically significant difference.

## 3. Results

### 3.1. PPAR*α* Inhibits Atherosclerotic Plaque Formation and Vascular Endothelial Injury

**Figure 1:**
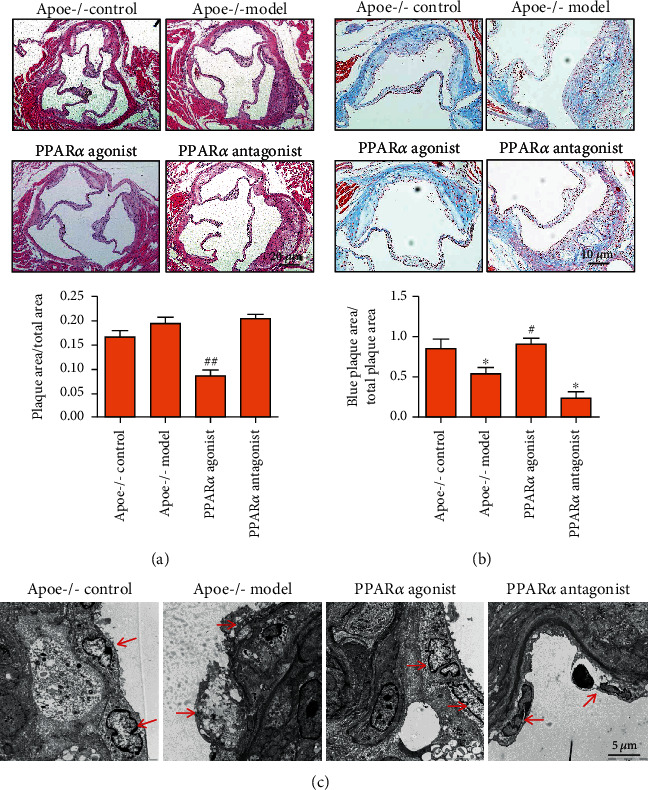
PPAR*α* inhibits atherosclerotic plaque formation and vascular endothelial injury. (a) HE staining showed that PPAR*α* agonists inhibited AS plaque formation. (b) Masson's trichrome staining showed that PPAR*α* agonists promoted plaque stability in the *Apoe*^−/−^ mouse model. (c) The PPAR*α* agonist made the surface of aortic plaque covered with intact endothelial cells and inhibited endothelial cell apoptosis and necrosis. ^∗^^, #^*P* < 0.05, ^##^*P* < 0.01, ∗: vs. control, #: vs. model. Scale bar, 5 *μ*m or 10 *μ*m or 20 *μ*m.

PPAR*α* is widely involved in lipid metabolism. Since the disturbance of lipid metabolism is an important factor leading to the formation of atherosclerotic plaque, PPAR*α* plays an important role in regulating atherosclerotic plaque formation. The current and our previous research found that PPAR*α* was involved in the repair of endothelial cell damage. Given that endothelial cell injury is the beginning of AS, we investigated how PPAR*α* affects the occurrence of AS. First, we showed that PPAR*α* agonists inhibited atherosclerotic plaque formation (Figure 1(a)) and played a role in promoting plaque stability in the *Apoe^−/−^* mouse model (Figure 1(b)). Then, we observed the effect of PPAR*α* on the aortic endothelial cell damage in *Apoe^−/−^* mice by transmission electron microscopy. Namely, the results showed that the endothelial cells in the plaques of the aortic tissue of the *Apoe^−/−^* model mice lost their basic cellular structure, and most of them were in the state of apoptosis and denudation. Endothelial cells were even missing in some plaques of the aortic tissue of *Apoe^−/−^* model mice. The PPAR*α* agonist ensured that the surface of the aortic plaque was covered with intact endothelial cells, and it inhibited endothelial cell apoptosis and necrosis. Intact endothelial cells inhibited monocyte adhesion and reduced the secretion of inflammatory factors, thereby preventing the formation of AS plaques. PPAR*α* antagonists played the opposite role and promoted endothelial cell apoptosis, necrosis, and denudation (Figure 1(c)). We speculated that PPAR*α* inhibited atherosclerotic plaque formation by protecting vascular endothelial cells.

### 3.2. PPAR*α* Inhibits Aging of Endothelial Cells in the Apoe^−/−^ Mouse Model

**Figure 2:**
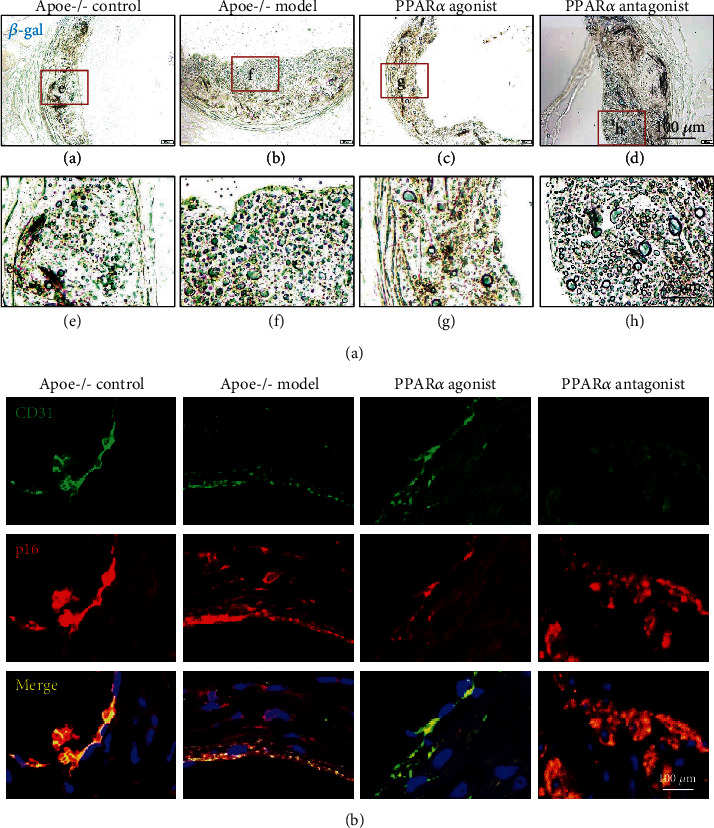
PPAR*α* inhibits aging of endothelial cells in the *Apoe^−/−^* mouse model. (a) *β*-gal staining showed that the number of senescent cells decreased after administration of the PPAR*α* agonist. (b) Immunofluorescence double labeling staining showed that there were a large number of aging endothelial cells in the atherosclerotic plaque of *Apoe^−/−^* mice, and the PPAR*α* agonist inhibited the denudation and maintained the integrity of aging endothelial cells in *Apoe^−/−^* model mice. Scale bar, 25 *μ*m or 100 *μ*m.

Aging caused by continued cell damage is the main pathological factor of AS. Therefore, *β*-gal staining was used to observe the aging cells in *Apoe^−/−^* mice plaques. We detected a large number of senescent cells in the left ventricular outflow plaques of *Apoe^−/−^* mice in each group. The senescent cells were distributed in different areas of the plaque, including the endothelial cell distribution area, foam cell distribution area, and smooth muscle cell distribution area, depending on the size of the atherosclerotic plaque and the pathological stage. The number of senescent cells in the atherosclerotic plaque also varied among the groups. Specifically, *Apoe^−/−^* model mice had a large number of senescent cells in the atherosclerotic plaque, but the number of senescent cells decreased after administration of the PPAR*α* agonist (Figure 2(a)). To further clarify whether PPAR*α* can inhibit vascular endothelial cell aging in *Apoe^−/−^* mice, the left ventricular outflow tract tissue sections of *Apoe^−/−^* mice were used to observe the aging of vascular endothelial cells. Endothelial cells were marked with CD31 (green), senescent cells were marked with p16 (red), and senescent endothelial cells (merged) were yellow. Although the results showed a large number of aging endothelial cells in the atherosclerotic plaque of *Apoe^−/−^* control mice, the basic morphology of senescent endothelial cells was still retained and the denudation of endothelial cells was not observed. However, besides the large number of senescent cells, intact vascular endothelial cells were not detected in *Apoe^−/−^* model mice, indicating that the senescent endothelial cells at the AS plaques of *Apoe^−/−^* model mice were denudated. The PPAR*α* agonist inhibited the denudation and maintained the integrity of aging endothelial cells in *Apoe^−/−^* model mice (Figure 2(b)). The results indicated that PPAR*α* participated in the development of AS by inhibiting the aging of vascular endothelial cells.

### 3.3. Expression of Aging-Related Proteins in the Apoe^−/−^ Mouse Aorta

**Figure 3:**
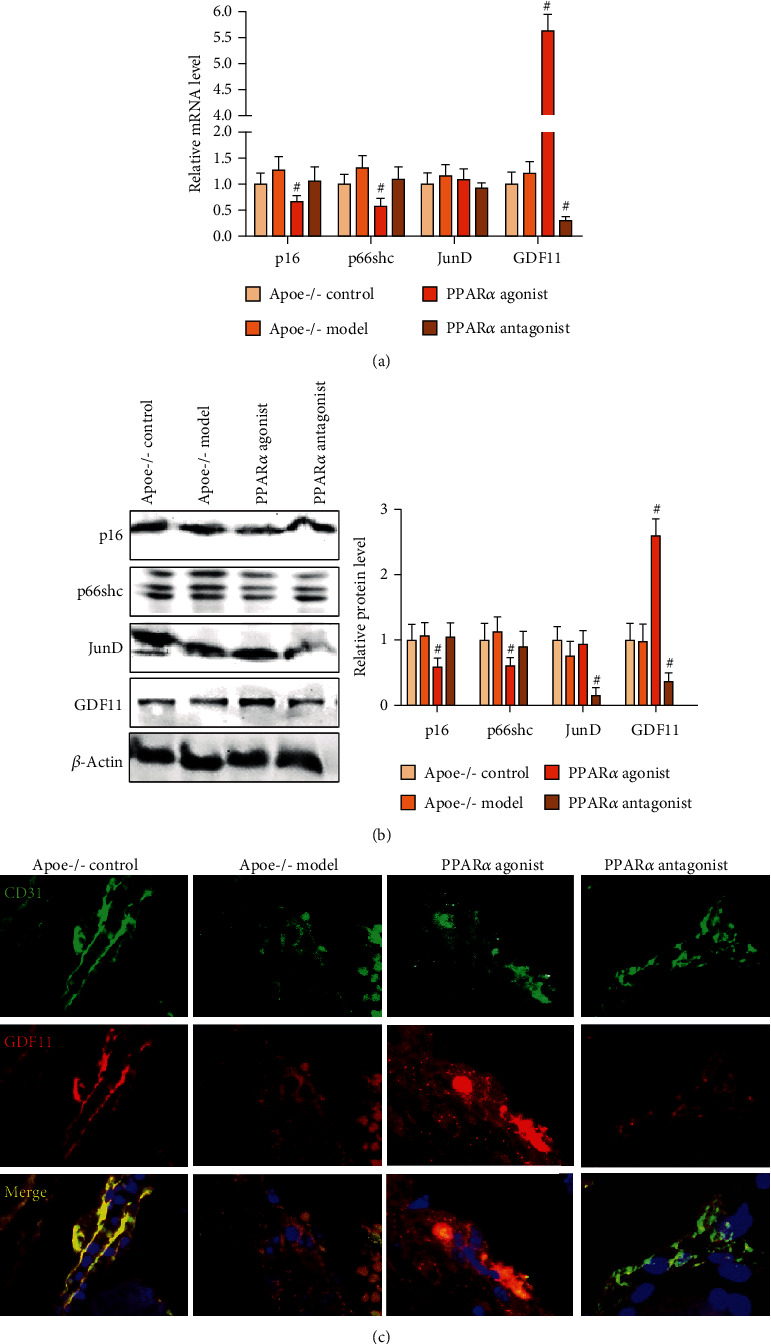
Expression of aging-related proteins in the *Apoe^−/−^* mouse aorta. (a) Real-time PCR showed that the PPAR*α* agonist significantly inhibited the expression of p16 and p66shc and promoted the expression of GDF11 at the mRNA level, while the PPAR*α* antagonist inhibited the expression of GDF11 at the mRNA level. (b) Western blot showed that the PPAR*α* agonist significantly inhibited the expression of p16 and p66shc and promoted the expression of GDF11 at the protein level. In contrast, the PPAR*α* antagonist inhibited the protein level of JunD and GDF11. (c) Immunofluorescence double labeling staining showed that the PPAR*α* agonist promoted the expression of GDF11, while the PPAR*α* antagonist inhibited the expression of GDF11 in vascular endothelial cells. ^#^*P* < 0.05, #: vs. model. Scale bar, 100 *μ*m.

Based on the existence of a large number of senescent cells in the atherosclerotic plaques of the left ventricular outflow tract of *Apoe^−/−^* mice, we detected the expression of aging-related proteins in the aortic tissue of *Apoe^−/−^* mice. Real-time PCR showed that the PPAR*α* agonist significantly inhibited the expression of p16 and p66shc and promoted the expression of GDF11 at the mRNA level. In contrast, the PPAR*α* antagonist inhibited the expression of GDF11 at the mRNA level (Figure 3(a)). Western blot showed that the PPAR*α* agonist significantly inhibited the expression of p16 and p66shc and promoted the protein level of GDF11 at the protein level, whereas the PPAR*α* antagonist inhibited the protein level of JunD and GDF11 (Figure 3(b)). Whether at the mRNA level or protein level, PPAR*α* significantly regulated the expression of GDF11. Interestingly, GDF11 expression declined with age in mice, and therefore, we further observed whether PPAR*α* delayed senescence of vascular endothelial cells by promoting GDF11 expression. The results showed that GDF11 was expressed in the vascular endothelial cells of the AS plaque of the left ventricular outflow tract in *Apoe^−/−^* mice. The expression of GDF11 was reduced in vascular endothelial cells of *Apoe^−/−^* model mice; however, the PPAR*α* agonist promoted the expression of GDF11, while the PPAR*α* antagonist inhibited the expression of GDF11 in vascular endothelial cells (Figure 3(c)). Therefore, we speculated that PPAR*α* might inhibit the aging of vascular endothelial cells in *Apoe^−/−^* model mice by promoting the expression of GDF11.

### 3.4. PPAR*α* Targeting GDF11 Participates in the Regulation of Vascular Endothelial Cell Senescence and AS

**Figure 4:**
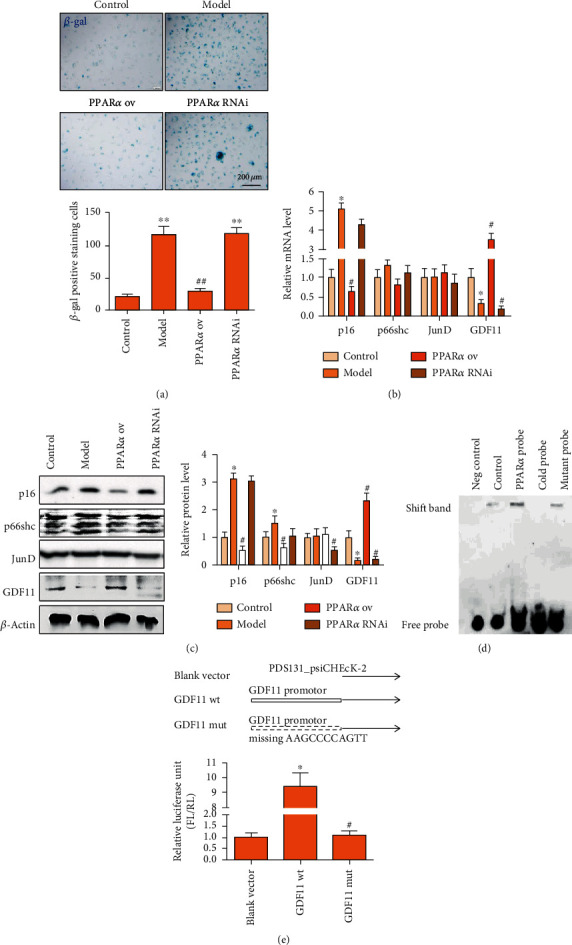
PPAR*α* targets GDF11 expression. (a) *β*-gal staining showed that overexpression of PPAR*α* significantly inhibited cell senescence. (b) Real-time PCR showed that PPAR*α* significantly inhibited the upregulation of p16 and the downregulation of GDF11 caused by ox-LDL exposure of cultured vascular endothelial cells (200 *μ*g/mL). (c) Western blot showed that the PPAR*α* agonist significantly promoted GDF11 expression and inhibited p16 and p66shc expression. (d) EMSA results showed that PPAR*α* bound to the labeled target probe, and partially to the mutant probe, indicating that PPAR*α* could bind to the GDF11 target sequence and participate in the regulation of downstream gene transcription. (e) Double-luciferase reporter gene experiment results indicated that PPAR*α* could bind to the GDF11-specific sequence. ^∗^^, #^*P* < 0.05, ^∗∗^^, ##^*P* < 0.01, ∗: vs. control, #: vs. model.

To further verify whether PPAR*α* inhibits vascular endothelial cell senescence by promoting GDF11 expression, HUVECs were used *in vitro* to observe the effect of PPAR*α* and GDF11 on endothelial cell function. First, PPAR*α* was overexpressed or interfered within HUVECs to observe their aging. The results showed that overexpression of PPAR*α* significantly inhibited cell senescence, while interference with PPAR*α* significantly promoted cell senescence (Figure 4(a)). Real-time PCR and western blot were used to detect the effect of PPAR*α* on the expression of senescence-related proteins in cultured vascular endothelial cells. The damage of ox-LDL to the cultured endothelial cells led to the upregulation of p16 and the downregulation of GDF11 at the mRNA level. PPAR*α* significantly inhibited the upregulation of p16 and the downregulation of GDF11 caused by ox-LDL (Figure 4(b)). At the protein level, p16 and p66shc were both upregulated, whereas GDF11 and junD were both downregulated in the cultured vascular endothelial cells subjected to ox-LDL. The expression of p66shc and JunD was not consistent with that of mRNA, which might suggest that PPAR*α* does not directly regulate the expression of p66shc and JunD (Figure 4(c)). On the basis of the results regarding the effect of PPAR*α* on the expression of aging-related proteins *in vivo* and *in vitro*, we further verified whether PPAR*α* directly targets GDF11 to regulate vascular endothelial cell senescence using EMSA and luciferase assays. The EMSA results showed that PPAR*α* bound to the labeled target probe, and partially to the mutant probe, indicating that PPAR*α* could bind to the GDF11 target sequence and participate in the regulation of downstream gene transcription (Figure 4(d)). Therefore, we constructed a reporter gene plasmid with a specific fragment of GDF11 inserted in front of the luciferase expression sequence (PDS131_psiCHECK-2-GDF11). The lentivirus GDF11 plasmid was introduced to EA.hy926 cells, and we observed whether the endogenous PPAR*α* can activate the GDF11 target promoter. Double-luciferase reporter gene experiment results indicated that PPAR*α* could bind to the GDF11-specific sequence (Figure 4(e)). Therefore, we concluded that PPAR*α* directly regulates the expression of GDF11 and influences the function of vascular endothelial cells.

**Figure 5:**
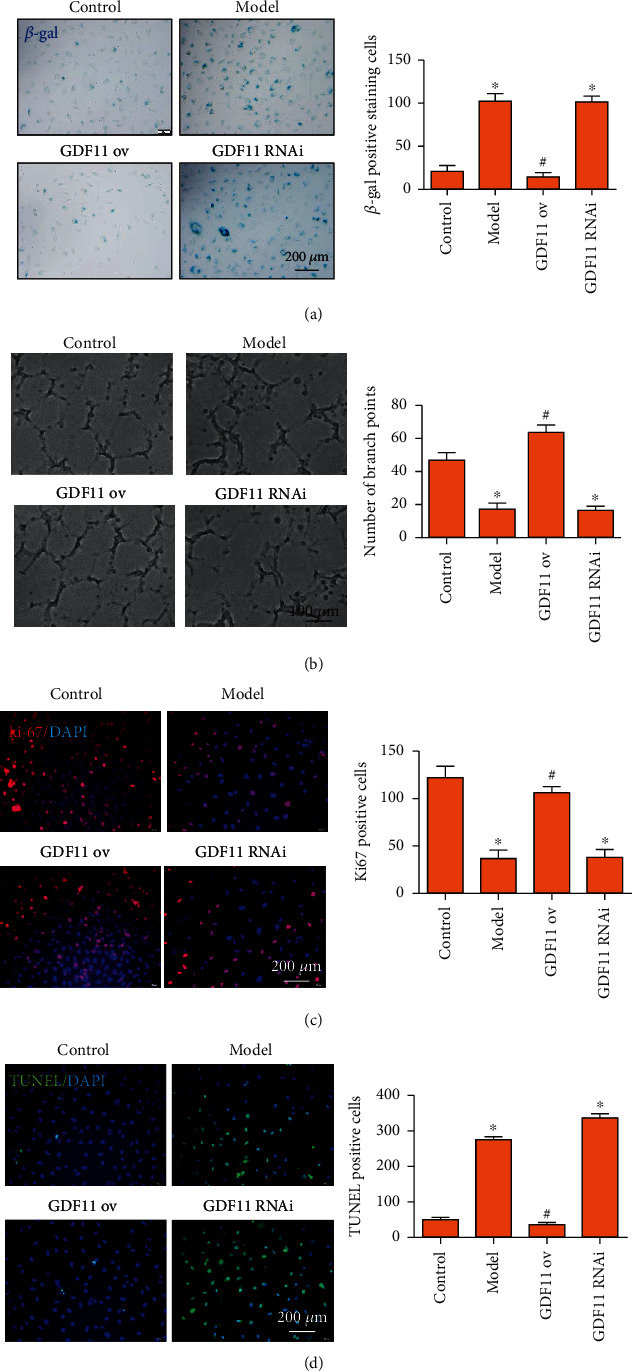
The effect of GDF11 on endothelial cell function. (a) *β*-gal staining showed that GDF11 inhibited vascular endothelial cell aging. (b) Angiogenesis assay showed that GDF11 improved vascular endothelial cell angiogenesis. (c) ki67 staining showed that GDF11 improved vascular endothelial cell proliferation. (d) TUNEL staining showed that GDF11 inhibited vascular endothelial cell apoptosis. ^∗^^, #^*P* < 0.05, ∗: vs. control, #: vs. model. Scale bar, 100 *μ*m or 200 *μ*m.

Of note, GDF11 reverses age-related cardiac hypertrophy and reduces neurodegenerative and neurovascular diseases. Furthermore, GDF11 also improves endothelial dysfunction, but there have been no studies to clarify the direct relationship between GDF11 and vascular endothelial cell aging and angiogenesis. To prove that PPAR*α* affects endothelial cell function by targeting GDF11, we first clarified the effect of GDF11 on endothelial cell aging. Here, we showed that GDF11 inhibited vascular endothelial cell aging and apoptosis, while it improved vascular endothelial cell proliferation and angiogenesis. Beta-gal staining showed that ox-LDL increased the number of senescent endothelial cells *in vitro*. The endothelial cells overexpressing GDF11 significantly reduced the number of senescent cells treated with ox-LDL (Figure 5(a)). The angiogenesis assay was used to detect the ability of endothelial cells to form blood vessels; the results indicated that the endothelial cells overexpressing GDF11 indeed rescued angiogenesis in ox-LDL treatment (Figure 5(b)). The role of GDF11 in promoting angiogenesis has a positive significance for vascular endothelial cell repair. Similarly, ki67 staining showed that overexpressing GDF11 significantly promoted endothelial cell proliferation despite ox-LDL treatment (Figure 5(c)). Finally, we examined the role of GDF11 in regulating apoptosis of vascular endothelial cells. ox-LDL induced apoptosis of a large amount of vascular endothelial cells, but vascular endothelial cells that overexpressed GDF11 avoided apoptosis caused by ox-LDL (Figure 5(d)). We speculated that the role of GDF11 in inhibiting aging and apoptosis and in promoting proliferation and angiogenesis of vascular endothelial cells has a positive effect on delaying the occurrence of AS.

**Figure 6:**
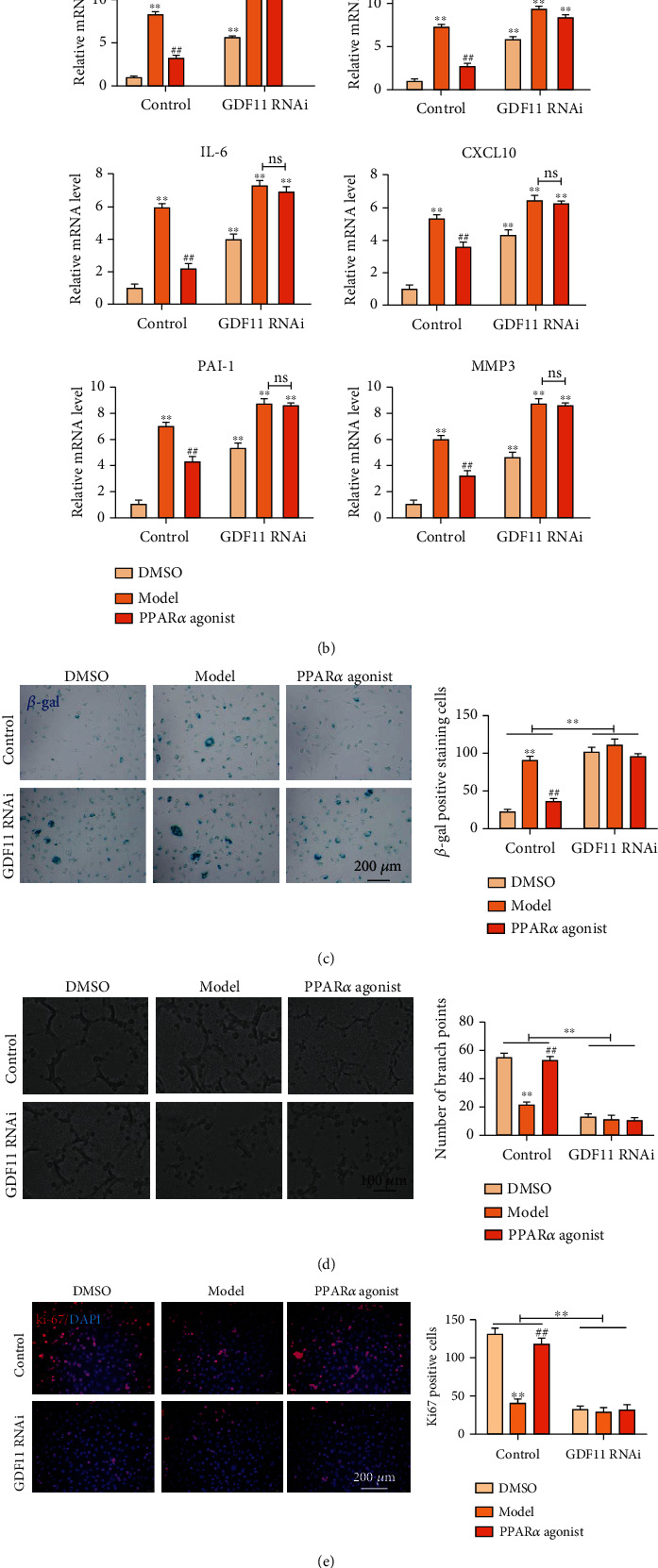
PPAR*α* influence on the function of vascular endothelial cells is achieved by regulating GDF11. (a) Western blot showed GDF11 expression while adding ox-LDL or PPAR*α* agonist to the stable GDF11-knockdown HUVECs. (b) Real-time PCR revealed a stark increment in SASP (including TNF*α*, IL-1*β*, IL-6, CXCL10, PAI-1, and MMP3) in HUVECs (with or without GDF11 knockdown) treated with ox-LDL. (c–f) When GDF11 expression was inhibited, the ability of PPAR*α* to promote HUVEC proliferation and angiogenesis was significantly reduced, while senescent and apoptotic cells increased significantly. ^∗∗^^, ##^*P* < 0.01, ∗: vs. control, #: vs. model. Scale bar, 100 *μ*m or 200 *μ*m.

To further investigate whether the influence of PPAR*α* on the function of vascular endothelial cells was achieved by regulating GDF11, we examined the aging phenotype while adding the PPAR*α* agonist to the stably transfected HUVECs with GDF11 interference. First, GDF11 expression was detected while adding ox-LDL or PPAR*α* agonist to the stable GDF11-knockdown HUVECs. ox-LDL inhibited GDF11 expression, while the PPAR*α* agonist enhanced GDF11 expression. In the stable GDF11-knockdown HUVECs with or without ox-LDL and PPAR*α* agonist, weak expression of GDF11 was detected (Figure 6(a)). Next, we examined the mRNA level of senescence-related secretory phenotype (SASP). RT-qPCR revealed a stark increment in SASP (including TNF*α*, IL-1*β*, IL-6, CXCL10, PAI-1, and MMP3) in HUVECs (with or without GDF11 knockdown) treated with ox-LDL. In the control group, the PPAR*α* agonist partially suppressed the expression of SASP, while in the GDF11 interference group, the PPAR*α* agonist lost the effect of inhibiting the expression of SASP (Figure 6(b)). These data suggested that PPAR*α* is involved in regulating cell senescence by targeting GDF11. To further clarify that PPAR*α* achieves the function of regulating vascular endothelial cell senescence by targeting GDF11, we examined the effects of PPAR*α* on aging, angiogenesis, proliferation, and apoptosis of HUVECs with GDF11 knockdown (Figures 6(c)–6(f)). We showed that when GDF11 expression was inhibited, the ability of PPAR*α* to promote HUVEC proliferation and angiogenesis was significantly reduced, while senescent and apoptotic cells increased significantly in these HUVECs. Based on the above data, we concluded that PPAR*α* inhibits senescence of vascular endothelial cells through upregulating the expression of GDF11 in HUVECs.

## 4. Discussion

Vascular endothelial cell senescence is closely related to AS, and our previous study found that PPAR*α* is related to the damage and repair of vascular endothelial cells and the occurrence of AS. In our research, we first predicted whether PPAR*α* targeted senescent-related proteins (p16, p21, p66shc, junD, sirt1, and GDF11) using online databases (http://jaspar.genereg.net/). The two best matching proteins were junD and GDF11. Therefore, we tested the expression of aging-related proteins in the aortic tissues of *Apoe*^−/−^ model mice which were given PPAR*α* agonists and antagonists and found that PPAR*α* agonists did not affect the expression of junD at the mRNA level but greatly promoted the expression of GDF11. Therefore, we profoundly explored whether PPAR*α* affects vascular endothelial cell senescence by regulating GDF11 expression. AS starts with the damage of vascular endothelial cells [9, 22]. Normal endothelial cells have a perfect mechanism of repairing damage, which can prevent endothelial cells from releasing cytokines and chemokines. In contrast, the senescent endothelial cells have a significantly reduced damage repair ability, which manifests in the release of a number of chemokines to promote monocyte adherence, aggregation, and migration to the intimal layer of blood vessels. Monocytes engulf oxidative lipids to form characteristic foam cells and promote the formation of AS plaques [23, 24]. Therefore, the prevention or repair of vascular endothelial cell damage is the primary factor to delay the occurrence of AS. PPAR*α* is a ligand-activated transcription factor of clinical interest as a drug target in various metabolic disorders, which also contributes to the activity of endothelial nitric oxide synthase (eNOS) and regulates vascular tone [12, 25]. PPAR*α* gene variants appear to be associated with higher plasma lipid levels and increased risk of coronary artery disease [26]. Although PPAR*α* has the function of promoting eNOS synthesis and repairing cell damage, novel PPAR*α* drugs with therapeutic potential for metabolic syndrome and cardiovascular diseases both target the lipid reduction. Some research reported that PPAR*γ* protects against vascular aging [27], but there were few studies on the correlation between PPAR*α* and aging. Our previous RNA sequence research found that PPAR*α* was involved in the regulation of damaged vascular endothelial cell repair [15]. The causes of endothelial cell aging mainly include cell replication aging and stress-induced cell premature senility. In addition to telomere shortening, the genetic factors, DNA damage, and oxidative stress are the causes of cell replication senescence. Stress-induced cell premature senility usually shows an aging phenotype within hours to days; therefore, failure to repair damaged cells in a timely and accurate manner is an important cause of cell aging. We speculated that the role of PPAR*α* in repairing damaged vascular endothelial cells was crucial for preventing senescence and AS.

GDF11 is a member of the TGF*β* superfamily with a controversial role in aging processes. The current evidence on GDF11 indicates a potential key role in reversing several pathological processes associated with aging, although the association with age-related AS is still unclear. Of note, it was shown previously that GDF11 is a circulating factor that reverses age-related cardiac hypertrophy [28]. Considering the existence of regenerative capacity of endogenous GDF11 in the peripheral tissues and brain, it will be important to investigate the mechanisms with therapeutic potential for a variety of age-related cardiovascular diseases [29–31]. However, the research of quantification of GDF11 in human aging and cardiovascular disease indicated that individuals with higher GDF11 were more likely to be frail, and higher GDF11 at surgical baseline was associated with rehospitalization and multiple adverse events [32]. It was reported that GDF11 induces loss of cardiac and skeletal muscle mass and function [33]; in contrast, the recent study indicated that exogenous GDF11 protected the heart from acute myocardial ischemia-reperfusion injury and suggested its role in promoting morphological and functional recovery in the early stage of myocardial ischemia-reperfusion injury [34]. Therefore, additional research is required to fully understand the extent of GDF11 influence on age-related changes under physiological and pathophysiological conditions. At present, there have been few studies on the effect of GDF11 on the function of vascular endothelial cells and AS. Using adenoassociated viruses-GDF11 and recombinant GDF11, it was found that GDF11 protects against endothelial injury and reduces atherosclerotic plaque in *Apoe*^−/−^ mice [35]. We overexpressed or interfered with GDF11 in primary HUVECs cultured *in vitro* and found that GDF11 inhibited cell senescence and apoptosis and promoted cell proliferation and angiogenesis. These effects have certain significance for inhibiting the occurrence of AS.

In summary, we demonstrated a likely causal role for PPAR*α* in vascular endothelial cell senescence and occurrence of AS, where PPAR*α* inhibited cell aging and plaque formation by directly targeting GDF11. Pharmacologic stimulation of PPAR*α* alleviated atherosclerotic plaque formation, vascular endothelial cell damage, and senescence, as well as increasing GDF11 expression in *Apoe*^−/−^ model mice. At the same time, we proved that PPAR*α* directly targeted the aging-related protein GDF11, thereby affecting the aging, proliferation, apoptosis, and angiogenesis of vascular endothelial cells *in vitro*. Our findings are consistent with the general hypothesis that inhibiting the aging of vascular endothelial cells helps prevent the formation of atherosclerotic plaques. Our work suggests that targeting PPAR*α* or senescent vascular endothelial cells could be a promising avenue for delaying, preventing, alleviating, or treating AS.

## 5. Conclusions

In conclusion, our results suggested that PPAR*α* inhibited vascular endothelial cell senescence and apoptosis and promoted vascular endothelial cell proliferation and angiogenesis by heightening GDF11 production, thereby delaying the occurrence of AS. We also suggested that vascular endothelial cell senescence was the key driver of AS formation. These novel findings provide a new highly promising strategy for the prevention of vascular endothelial cell senescence and AS.

## Data Availability

All data used to support the findings of this study were collected in accordance with the scientific research criteria and are available from the corresponding author upon request.
